# Clinical risk factors associated with postoperative delirium and evaluation of delirium management and assessment team in lung and esophageal cancer patients

**DOI:** 10.1186/s40780-014-0002-3

**Published:** 2015-01-28

**Authors:** Kiminaka Murakawa, Yoshihisa Kitamura, Saori Watanabe, Shiho Hongo, Kazuaki Shinomiya, Toshiaki Sendo

**Affiliations:** Department of Pharmacy, Okayama University Hospital, 2-5-1 Shikata-cho, Okayama, 700-8558 Japan; Department of Clinical Pharmacy, Graduate School of Medicine, Dentistry and Pharmaceutical Sciences, Okayama University, 2-5-1 Shikata-cho, Kita-ku, Okayama 700-8558 Japan

**Keywords:** Delirium, Postoperative patients, Medical team, Risk factors

## Abstract

**Background:**

Delirium is an acute change in cognition and concentration that complicates the postoperative course. Patients who suffer delirium after surgery have an increased risk of persistent cognitive impairment, functional decline, and death. Postoperative delirium is also associated with an increased length of hospital stay and higher costs. With the goal of preventing delirium in postoperative patients, we organized a medical team from the Delirium Management and Assessment Center (D-mac) at Okayama University Hospital in January 2012. The team members consisted of physicians, pharmacists, nurses, and clinical psychologists.

**Methods:**

We retrospectively reviewed the medical records of patients with delirium to examine risk factors related to the patients’ background.

**Results:**

Fifty-nine postoperative patients with lung or esophageal cancer were investigated; 25% exhibited delirium during hospitalization. Multiple logistic regression analysis showed significant associations between the presence of delirium and a past history of delirium (odds ratio, 4.22; 95% CI, 1.10-16.2; p = 0.09) and use of benzodiazepine receptor agonists (odds ratio, 3.97; 95% CI, 1.09-14.5; p = 0.03). Intervention by the D-mac significantly reduced the rate of delirium episodes in lung cancer patients (p =0.01). Notably, prior to intervention, the incidence of delirium was 100% when three high-risk factors for delirium were present. In contrast, the incidence of delirium in patients with three high-risk factors decreased following implementation of the D-mac intervention.

**Conclusions:**

These findings suggest that active participation by various staff in the medical team managing delirium had a marked therapeutic impact.

## Background

Delirium is a disturbance of consciousness characterized by changes in cognition, perceptual disturbances, and a reduced ability to focus, sustain, or shift attention [1]. These symptoms typically manifest in disorientation, memory impairment, and alteration of mental processes, which can present as a hyperactive form, a hypoactive form, or a combination of the two [2,3]. Delirium has been associated with an increase in the length of hospital stay and complication rates, as well as functional decline [3,4]. Several studies have investigated risk factors such as advanced age, preoperative cognitive impairment, cephalopathy and delirium events, and alcoholism [3,5,6].

Surgery itself may play a role in delirium [3]. Postoperative delirium, a common and serious complication following surgery, has been associated independently with prolonged hospital stays, increased costs, and higher mortality [7].

**Figure 1:**
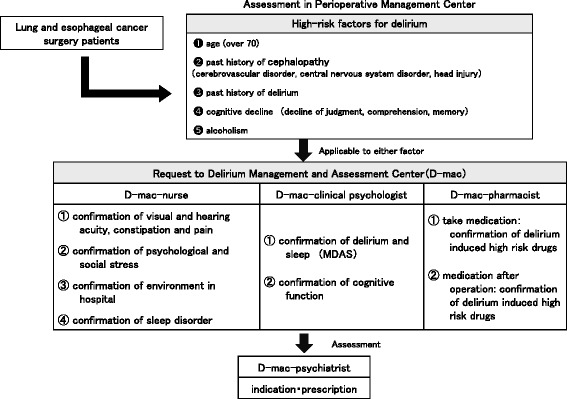
**Perioperative management at the Okayama University Hospital.** The Perio team (physicians, pharmacists, nurses) actively participated in the safe perioperative management of lung and esophageal cancer patients and screened for patients who had a high risk of delirium. The D-mac was composed of physicians, pharmacists, nurses, and clinical psychologists. The D-mac has actively participated in supporting surgical patients at high risk of delirium.

Given this background, physicians, pharmacists, nurses and clinical psychologists at Okayama University Hospital (Okayama, Japan) formed a Delirium Management and Assessment Center (D-mac), which has actively participated in supporting surgical patients since January 2012. Additionally, the hospital established a Perioperative Management Center (Perio). The Perio team (physicians, pharmacists, nurses) actively participated in the safe perioperative management of lung and esophageal cancer patients and screened for patients who had a high risk of delirium. We defined the high-risk factors for delirium as age (over 70), past history of cephalopathy, past history of delirium, cognitive decline, and alcoholism [3,5,6]. The Perio then referred these high-risk patients to the D-mac (Figure 1).

In general, delirium-inducing factors are categorized as predisposing factors, promoting factors, or direct factors [8]. Pharmaceuticals are considered a direct factor in the development of a delirium episode [8]. As a prophylactic intervention against delirium, the pharmacists in charge of the D-mac mainly evaluated any medicines taken by the patients as well as postoperative medications.

In the present study, we identified the predictive factors (age (over 70), past history of cephalopathy, past history of delirium, cognitive decline, and alcoholism) for delirium by examining the relationship between baseline patient characteristics and delirium in patients with postoperative-induced delirium, particularly those with lung and esophageal cancer. Furthermore, we configured the clinical end point for the intervention of the D-mac to be the incidence of delirium in this study. To investigate the impact of intervention by the D-mac, we examined the frequency of delirium episodes before and after participation by the D-mac.

## Methods

### Patient characteristics

Data were collected retrospectively on 119 patients with delirium in our hospital during the period from 1 January, 2011, to 31 December, 2011. We researched age, sex, the presence or absence of surgical intervention, and the duration of delirium. Delirium was diagnosed postoperatively by the attending physician based on the criteria of the Diagnostic and Statistical Manual of Mental Disorders IV edition, Text Revision (DSM-IV-TR), which are regarded as the most inclusive criteria for delirium. The study was conducted in accordance with the ethics committee of the Graduate School of Medicine, Dentistry and Pharmaceutical Sciences, Okayama University.

### Effects of delirium-inducing risk factors in postoperative patients with lung or esophageal cancer

Data on 59 preoperative patients with lung or esophageal cancer at our hospital during the period from 30 June, 2011, to 1 January, 2012, were collected retrospectively. We researched age, sex, disease (lung or esophageal cancer), 5 high-risk factors for delirium (age >70 years, past history of cephalopathy with cerebrovascular disorder, past history of delirium, cognitive decline, and alcoholism), and sleep disorder as the primary symptom of delirium [9]. Furthermore, we examined the utilization of 8 classifications of high-risk drugs, as follows: (1) benzodiazepines, (2) H2 blockers, (3) antihistamine drugs, (4) steroids. (5) anti-cholinergic drugs with tricyclic and tetracyclic anti-depressants, (6) anti-epileptic drugs, (7) anti-parkinson drugs, and (8) opioids.

### Effects of D-mac interventions in postoperative lung cancer patients

The present D-mac-implemented program was initially adopted by our hospital on 1 January, 2012. Data were collected retrospectively on 88 preoperative patients with lung cancer for the period from 1 January, 2011, to 31 December, 2011, prior to implementation of the D-mac interventions; 99 preoperative patients with lung cancer who were treated from 1 January, 2012, to 31 December, 2012, provided data for the period following implementation of the D-mac intervention. We researched age, sex, disease, the presence or absence of the five high-risk factors for delirium (age > 70 years, past history of cephalopathy with cerebrovascular disorders, past history of delirium, cognitive decline, and alcoholism).

### Statistics

Categorical variables were compared using the χ^2^ test. The association between risk factors and the occurrence of delirium was assessed using multivariate logistic regression and expressed in terms of the odds ratio (OR) and 95% confidence interval (CI). Sex, age, past history of cephalopathy with cerebrovascular disorders, past history of delirium, cognitive decline, alcoholism, sleep disorders, and medications served as covariates. Statistical significance was defined as p <0.05.

## Results

### Patient background

**Figure 2:**
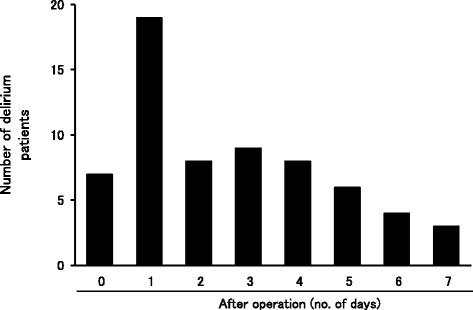
**Time-dependent frequency of delirium patients after surgery.** We investigated that 76 patients had delirium following an operation. The mean duration of delirium episodes was 5.1 days.

Table 1 shows the incidence of delirium in the operative patients. In total, 119 patients with delirium (mean age 70.9 years [range, 32–89 years]) were included in this study. Of these, 76 patients had delirium following an operation. The mean duration of delirium episodes was 5.1 days (range, 0–59 days). Furthermore, delirium more often occurred within 3 days versus within 4 days after surgery (Figure 2, Table 2). Compared with patients exhibiting delirium within 3 days, those exhibiting delirium after 4 days were on average younger (73.8 versus 65.8 years, respectively).

**Table 1:** **Background of postoperative delirium patients**

Number of patients	119
Sex (male female)	86:33
Mean age	70.2 (32-89)
Number of postoperative delirium patients	76
Durations of delirium events (days)	5.1 (0-59)

**Table 2:** **Background of period of postoperative induced delirium**

		**After operation (<3 days)**	**After operation (> days)**
Number of patients		43	33
Sex	Male	31	24
	Female	12	9
Mean age		43	33

### Effects of delirium-inducing factors in postoperative lung and esophageal cancer patients

Table 3 summarizes the baseline characteristics of 59 preoperative patients with lung or esophageal cancer. The mean age was 74.6 years (range, 44–87 years). A greater proportion of patients had lung cancer (71%) than esophageal cancer (29%). Additionally, more than half (n = 36; 61%) of these patients had been treated with drugs known to increase the risk of delirium. Delirium was diagnosed in 15 of the 59 (25%) patients during the study period.

**Table 3:** **Background of postoperative delirium of lung and esophageal cancer patients**

	**Number**		**%**
Number of patients	59		
Sex (male female)	41:18		69:31
Mean age	74β (44-87)		
Lung cancer: esophageal cancer	42:17		71:29
Delirium inducing high-risk factors	Number		%
	Yes	No	
Past history of cephabpathy	22	37	37:63
Past history of delirium	12	47	20:80
Cognitive decline	11	44	20:80
Alcoholism	8	51	14:86
Sleep disorder	17	42	29:71
High-risk drugs	36	23	61:39
Postoperative delirium events	Number		%
Number of postoperative delirium	15		25
Sex (male: female)	12:3		80:20

Of the high-risk drugs for delirium, nearly half (49%) of the patients used benzodiazepine receptor agonists (49%) (Table 4). Patients also took H2 blockers (17%), antihistamines (12%), and steroids (7%).

**Table 4:** **Delirium-inducing high-risk drugs**

**High-risk drungs**	**Number**	**Taking medication (%)**
Benzodiazepines	29	49
H2 blockers	10	17
Antihistamine drugs	7	12
Steroids	4	7
Anticholinergic drugs (tricyclic and tetracyclic antidepressant)	3	5
Antiepileptic drugs	3	5
Antiparkinson drugs	1	2
Opioids	0	0

Tables 5 through 7 present the results of the logistic regression analysis. A past history of delirium and the use of benzodiazepines significantly increased the odds of postoperative delirium (OR 4.22, 95% CI, 1.10-16.2; p = 0.03 for past history of delirium; OR 3.97, 95% CI, 1.09-14.5; p = 0.03 for benzodiazepine use) (Tables 5 and 6). Use of etizolam was also significantly associated with delirium (p = 0.02) (Table 7).

**Table 5:** **Relationships between patient background and postoperative delirium events**

	**Odds ratio**	**95% CI**	**P-values**
Sex (male)	2.07	0.50-8.48	0.30
Age (>70)	3.11	0.36-27.2	0.28
Lung cancer	1.87	0.45-7.69	0.38
Past history of cephalopathy	1.69	0.51-5.56	0.38
Past history of delirium	4.22	1.10-16.2	0.03
Cognitive decline	3.24	0.80-13.1	0.09
Alcoholism	0.97	0.17-5.44	0.97
Sleep disorder	2.00	0.58-6.90	0.09

**Table 6:** **Relationship between high-risk drugs and postoperative delirium events**

**High-risk drugs**	**Odds ratio**	**95% CI**	**P-values**
Benzodiazepines	3.97	1.09-14.5	0.03
Anticholinergic drugs (tricyclic and tetracyclic antidepressant)	1.50	0.13-17.8	0.75
H2 blockers	1.32	0.29-5.93	0.71
Steroids	1.32	0.29-5.93	0.24
Antihistamine drugs	0.45	0.05-4.10	0.47

**Table 7:** **Relationship between benzodiazepines and postoperative delirium events**

**Drugs**	**Number**	**%**	**Odds ratio**	**95% CI**	**P-values**
Brotizolam	12	41	1.63	0.41-6.49	0.48
Zolpidem	9	31	1.58	0.34-7.31	0.55
Etizolam	4	17	10.8	1.02-112.9	0.02

### Effect of D-mac management on delirium in postoperative patients with lung cancer

**Figure 3:**
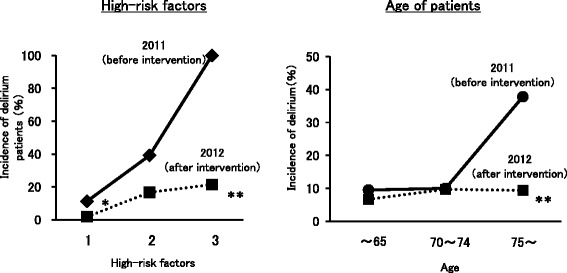
**Correlation between incidence of delirium and high-risk factors and age with or without D-mac intervention.** We investigated the association of delirium episodes with hig-risk factors and age with or without D-mac intervention. Values are expressed as the incidence of delirium patients (%). *p < 0.05, **p < 0.01, significantly different from 2011 (before D-mac intervention).

During the period from January 2011 to December 2012, the Perio referred 187 patients to the D-mac (Table 8). Since January 2012, the D-mac has actively participated in managing surgical patients with lung and esophageal cancer. Of the 187 patients, 88 had received intervention without D-mac management between January and December 2011. The remaining 99 patients had received intervention with D-mac management commencing in 2012. Between 2011 and 2012, the characteristics of the patients did not change in terms of age, past history of cephalopathy or delirium, cognitive decline, and alcoholism. However, the number of patients with delirium significantly decreased after the implementation of D-mac interventions in 2012. Furthermore, in 2011, the incidence of delirium rose to 100% in patients who had three high-risk factors. However, the incidence of delirium fell to 21.4% following intervention by the D-mac (Figure 3). In patients over 75 years, delirium events occurred in 37.8% of postoperative patients with lung cancer. However, the incidence of delirium decreased to 9.4% with D-mac intervention (Figure 3).

**Table 8:** **Background of postoperative delirium in** **lung patients**

		**Before intervention (2011)**		**After intervention (2012)**		**P-values**
		**Number**	**%**	**Number**	**%**	
High-risk patients		88	47.0	99	53.0	
Delirium patients		19	21.6	9	9.10	0.01
Sex	Male	57	64.7	63	63.6	0.87
	Female	31	35.3	36	36.4	
Mean age		71.9 (30-88)		73.8 (34-87)		0.16
Age(>70)		67	76.1	84	84.8	0.13
Past history of cephalopathy		26	29.5	28	28.3	0.85
Past history of delirium		10	11.4	19	19.2	0.14
Cognitive decline		5	5.7	9	9.1	0.38
Alcoholism		10	11.4	15	15.2	0.45

## Discussion and conclusions

The present study demonstrated that team medical care-implemented pharmaceutical management for the patients contributed to a prevention in surgical patients with lung and esophageal cancer. Furthermore, the active participation of pharmacists in the cases of surgical patients with lung and esophageal cancer contributed to a decrease in the incidence of delirium through pharmaceutical care.

In general, delirium is characterized by symptoms of disturbance in consciousness, such as disorientation, hallucinations, and delusion [10]. Delayed improvement in illness due to the aggravation of delirium has been reported [11]. Early discovery of delirium and intervention are desirable as one-third of delirium cases can be prevented through the identification of high-risk patients and appropriate intervention [12].

Causes of delirium are broadly categorized as predisposing, promoting, and direct factors. In this study, many patients with delirium developed this condition after surgery. As such, postoperative delirium has been reported in about 40% of surgical patients [13,14]. ‘Surgery’ is considered a predisposing factor for delirium [3] and influences physical causes, such as pain, mental causes, such as postoperative anxiety and depression, and promoting factors, such as environmental changes in an intensive care unit. The onset of postoperative delirium occurred relatively early following surgery, as previously reported, and this tendency for early onset was stronger in more elderly patients [15,16]. This suggests that elderly surgical patients may bear a higher risk for delirium and may therefore require early intervention before surgery. In our study, the incidence of delirium was higher in older patients for several days immediately following surgery.

The D-mac at our hospital identified several high-risk factors for delirium. Of these, ‘past medical history of delirium’ was shown to be a significant risk factor for postoperative delirium. The presence of a past medical history of delirium was the only significant independent high-risk factor. However, the incidence of delirium rose when multiple high-risk factors were present, suggesting the importance of confirming the presence of multiple high-risk factors in patients before surgery. In fact, the incidence of delirium rose to 100% in patients who had three high-risk factors before D-mac intervention in surgical patients in this study.

‘Drugs’ are considered to be a ‘direct factor’ causing delirium [6], and represent causative factors for delirium in many elderly patients [17,18]. The prevalence of insomnia has recently increased and is particularly high in the elderly [19]. Accordingly, commensurate with the aging of the population in Japan, the prescription rate for benzodiazepine receptor agonists has increased [20]. Benzodiazepine receptor agonists are well known for inducing delirium [21-23]; these drugs were most frequently brought in by patients on admission in this study. The incidence of postoperative delirium was significantly higher in patients who were medicated with these drugs. Many benzodiazepine receptor agonists are mainly metabolized in the liver [24]. Liver and kidney functions are generally reduced in the elderly, particularly after surgery [13,25]. Moreover, absorption of benzodiazepine receptor agonists is influenced by intestinal peristalsis, but this function is reduced after surgery in many cases. Therefore, the ability to metabolize benzodiazepine receptor agonists may be markedly reduced postoperatively in elderly patients. The reduced metabolism and changes in absorption may promote drug accumulation and sensitivity and induce cognitive dysfunction, a known effect specific to benzodiazepine receptor agonists. Given these mechanisms, benzodiazepine receptor agonists are likely to cause postoperative delirium in elderly patients. Attention should be given to patients who are treated with these drugs prior to surgery. Brotizolam, zolpidem, and etizolam accounted for a large proportion of the benzodiazepine receptor agonists brought to the hospital by patients on admission. In particular, etizolam was significantly associated with the development of postoperative delirium. This finding suggests that etizolam should be administered to postoperative elderly patients with extreme caution. Moreover, of the three drugs, etizolam is the most lipid-soluble (distribution coefficient: 354, pH 7, octanol/water) and exhibits high potency. Benzodiazepine receptor agonists were most frequently brought in by patients on admission. However, it was not obvious whether delirium occurred under conditions in which benzodiazepine receptor agonists were taken or not taken in this retrospective study. Further study is underway to clarify this point. For patients complaining of insomnia and at risk for postoperative delirium, the D-mac currently recommends prescribing trazodone hydrochloride, which, among the triazolopyridine derivatives, exerts a strong hypnotic effect but does not act on the benzodiazepine receptor. However, the usefulness of trazodone hydrochloride in preventing postoperative delirium should be further investigated.

The incidence of postoperative delirium was significantly reduced by the D-mac intervention team comprised of psychiatrists, pharmacists, nurses, and clinical psychologists. This demonstrates that early identification of high-risk factors and drugs potentially inducing delirium along with appropriate interventions are beneficial in preventing postoperative delirium. Since delirium may develop when multiple risk factors are concomitantly present [17,26,27], a comprehensive approach involving the collaboration of an interdisciplinary team may be effective.

On the other hand, the role of the ward pharmacists is to evaluate the patient’s background characteristics and medications brought from home and to formulate appropriate recommendations based on these assessments. When reviewing a patient’s medications on admission, it is essential that ward pharmacists identify drugs associated wuth a high risk of delirium and advise the attending physicians to withdraw these drugs and prescribe alternative medications. Expanding and developing the pharmacist’s role in this regard may assist in improving the quality of medical care aimed at preventing delirium. In turn, these measures may benefit the patient, and thereby achieve an important goal of ward drug management.
